# Metastatic clear cell renal carcinoma – an unusual response to Temsirolimus in second line therapy

**Published:** 2016

**Authors:** DL Stanculeanu, A Lazescu, DD Zob, R Bunghez, R Anghel, TD Poteca

**Affiliations:** * Institute of Oncology, Bucharest, Romania

**Keywords:** clear cell, renal, metastatic, metastatic

## Abstract

Renal cell carcinoma (RCC) represents 3% of all cancers, with the highest incidence occurring in the most developed countries and representing the seventh most common cancer in men and the ninth most common cancer in women. The understanding of the tumor molecular biology and the discovery of new drugs that target molecular pathways have increased the arsenal against advanced renal cell carcinoma and improved the outcomes in the patients suffering from these affections. Studying the molecular signaling that controls the tumor growth and the progression has led to the development of molecular therapies targeting the vascular endothelial growth factor (VEGF) and mammalian target of rapamycin (mTOR) pathways, resulting in a significant improvement in the overall survival and quality of life. Sunitinib represents an inhibitor of VEGFR 1-3, c-kit, FLT-3 and PDGFR.

We present the case of a patient with metastatic clear cell RCC with a treatment effect following sequential VEGF and mTOR inhibitor treatment. Under sunitinib treatment, the patient had a progression free survival (PFS) of approximately 9 months, similar to the PFS observed in clinical trials. Sunitinib was well tolerated by this patient. Temsirolimus, an mTOR inhibitor, is currently only approved for the first-line treatment of mRCC patients with poor prognosis. This study analyzes a treatment effect of second line temsirolimus in a patient with metastatic renal cell carcinoma (mRCC).

## Introduction

**Fig. 1:**
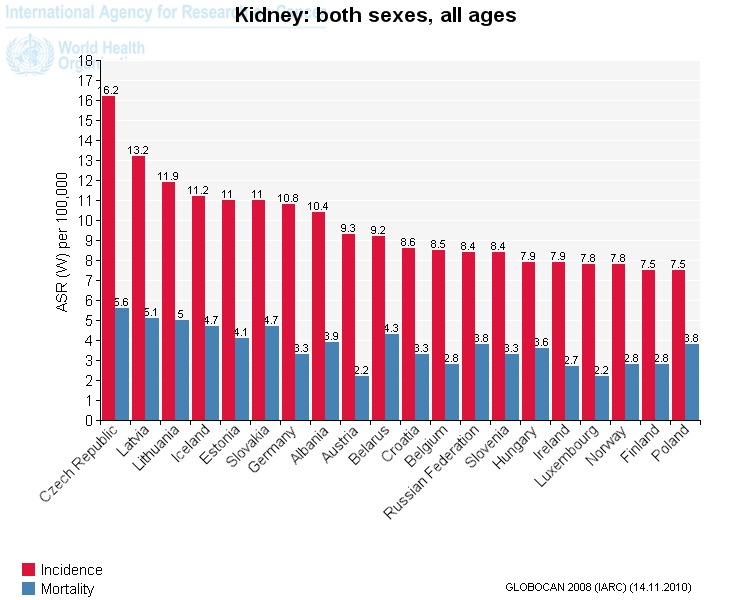
Incidence of renal cell cancer

Renal cell carcinoma (RCC) represents 3% of all cancers, with the highest incidence occurring in the most developed countries and representing the seventh most common cancer in men and the ninth most common cancer in women. The median age at diagnosis is 65 years and 25% of the patients present with an advanced stage of the disease. Of all kidney tumors, 85% are RCC and the risks factors for disease are obesity, smoking, family history. The estimated average 5-years survival rates in RCC according to the stage of the disease is the following: Stage I: 96%; Stage II: 82%; Stage III: 64%; Stage IV: 23%. There are ~209 000 new cases and 102 000 deaths per year worldwide. Approximately 2%–3% of RCC are hereditary and several autosomal dominant syndromes are described, each with a distinct genetic basis and phenotype, the most common one being Von Hippel Lindau disease. Conventional chemotherapy is associated with poor outcomes in metastatic renal cell carcinoma [**1**] (**Fig. 1**).

**Renal Cell Cancer (incidence and mortality) in Europe (Globocan 2012)**

**Fig. 2:**
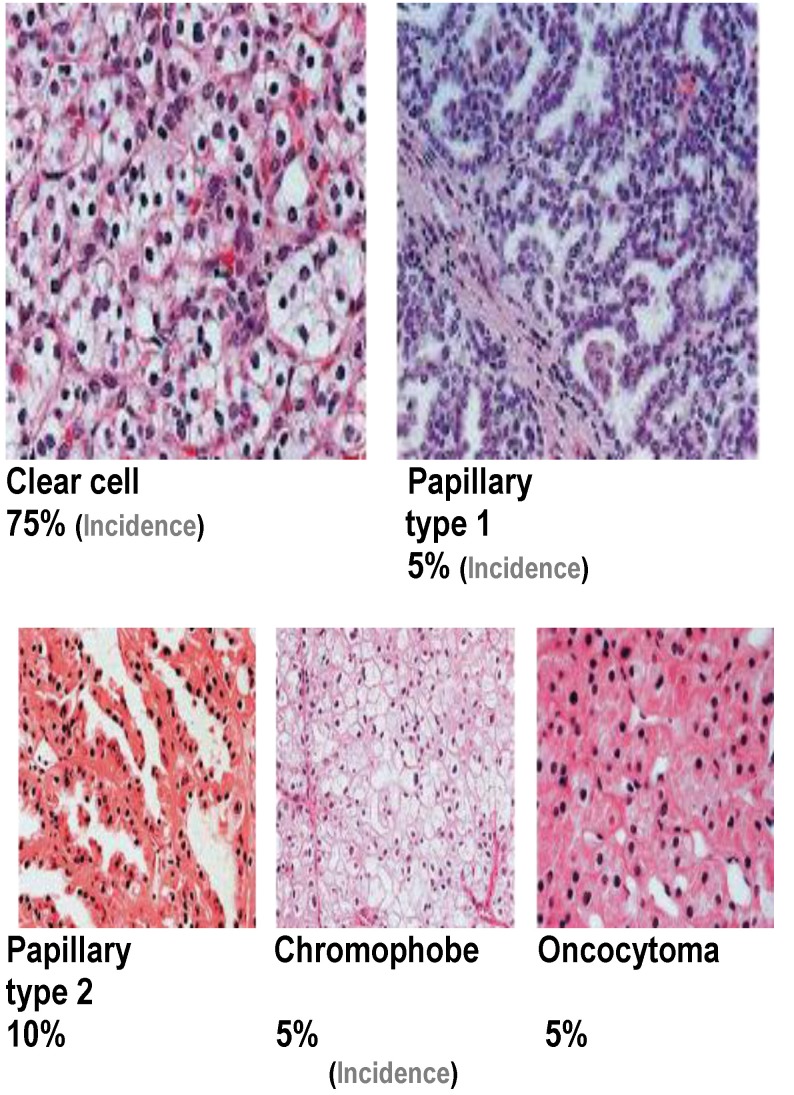
Histological types of renal cell carcinoma (Linehan WM et al. J Urol. 2003)

The understanding of tumor molecular biology and the discovery of new drugs that target molecular pathways have increased the arsenal against the advanced renal cell carcinoma and improved the outcomes in these patients. Studying the molecular signaling that controls tumor growth and progression has led to the development of molecular therapies targeting the vascular endothelial growth factor (VEGF) and mammalian target of rapamycin (mTOR) pathways, resulting in a significant improvement in the overall survival and quality of life. Sunitinib represents an inhibitor of VEGFR 1-3, c-kit, FLT-3 and PDGFR. Sunitinib has antitumor and antiangiogenic activity and was approved by the FDA in 2006, being now considered a first-line therapy for mRCC. It is orally administered with the recommended daily dose of 50 mg/ daily by a schedule 4w/ 2w until the disease progression [**2**]. Mammalian target of rapamycin (mTOR) is critical for cellular growth, proliferation, and angiogenesis in clear cell renal carcinoma. This pathway is more significantly mutated in high-grade tumors, and tumors with poor prognostic features. The novel therapeutics targeting the mTOR pathway approved by the FDA in the treatment of metastatic renal carcinoma is temsirolimus and everolimus. RCC is a heterogeneous group of diseases, different tumors characterized by distinct histology, natural histories, and responses to therapy [**3**] (**Fig. 2**).

## Case presentation

**Fig. 3:**
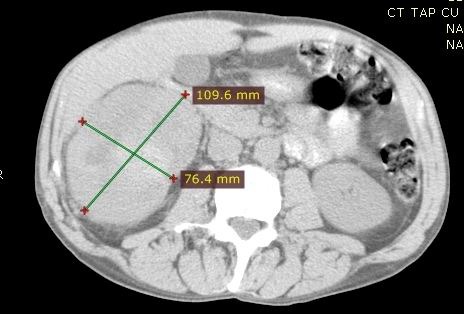
CT scan of the abdomen

We present the case of a patient with metastatic clear cell RCC with a treatment effect following sequential VEGF and mTOR inhibitor treatment. The patient’s characteristics are the following: age 50 years old, gender - male with no relevant personal medical history. At the moment of the diagnosis the patient presented right lumbar pain resistant to analgesia and macroscopic hematuria. In February 2011, the patient performed a CT scan, which revealed a mass in the right kidney. Thoracic CT scans showed no pulmonary metastases and bone scan showed no secondary lesions (**Fig. 3**).

**Fig. 4:**
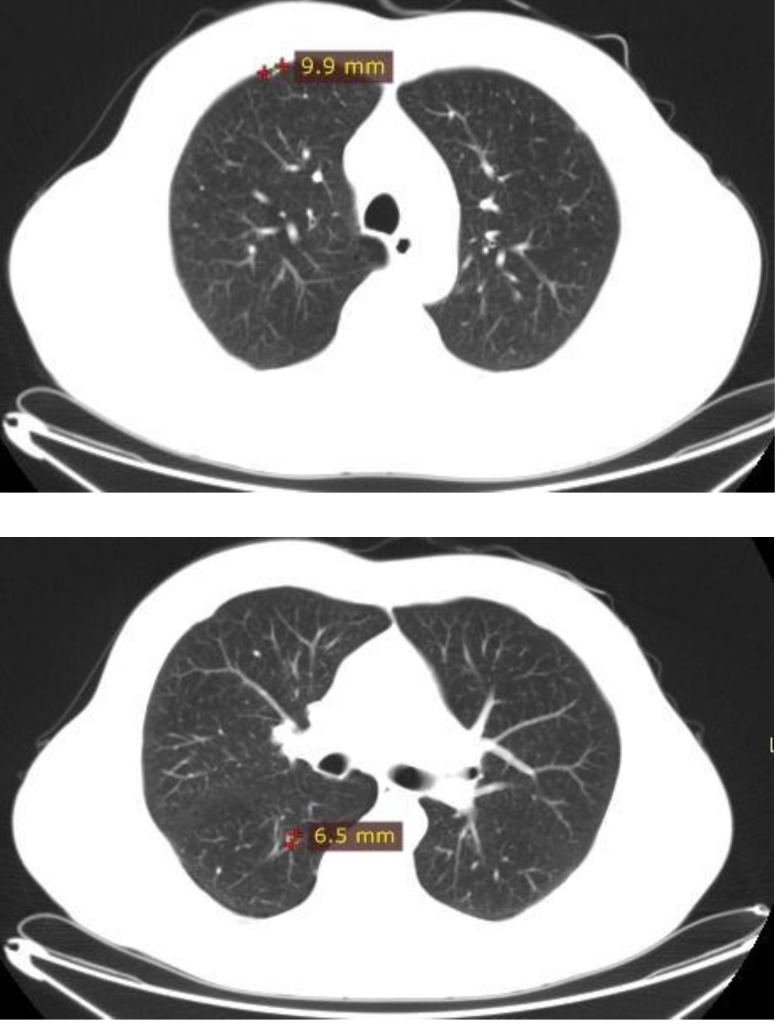
Thoracic CT scan

Nephrectomy was performed in March 2011 and the histological examination indicated a clear cell carcinoma RCC (pT3apN0), with perirenal fat extension. In January 2012, the routine CT scan revealed pulmonary metastases (**Fig. 4**).

**Fig. 5:**
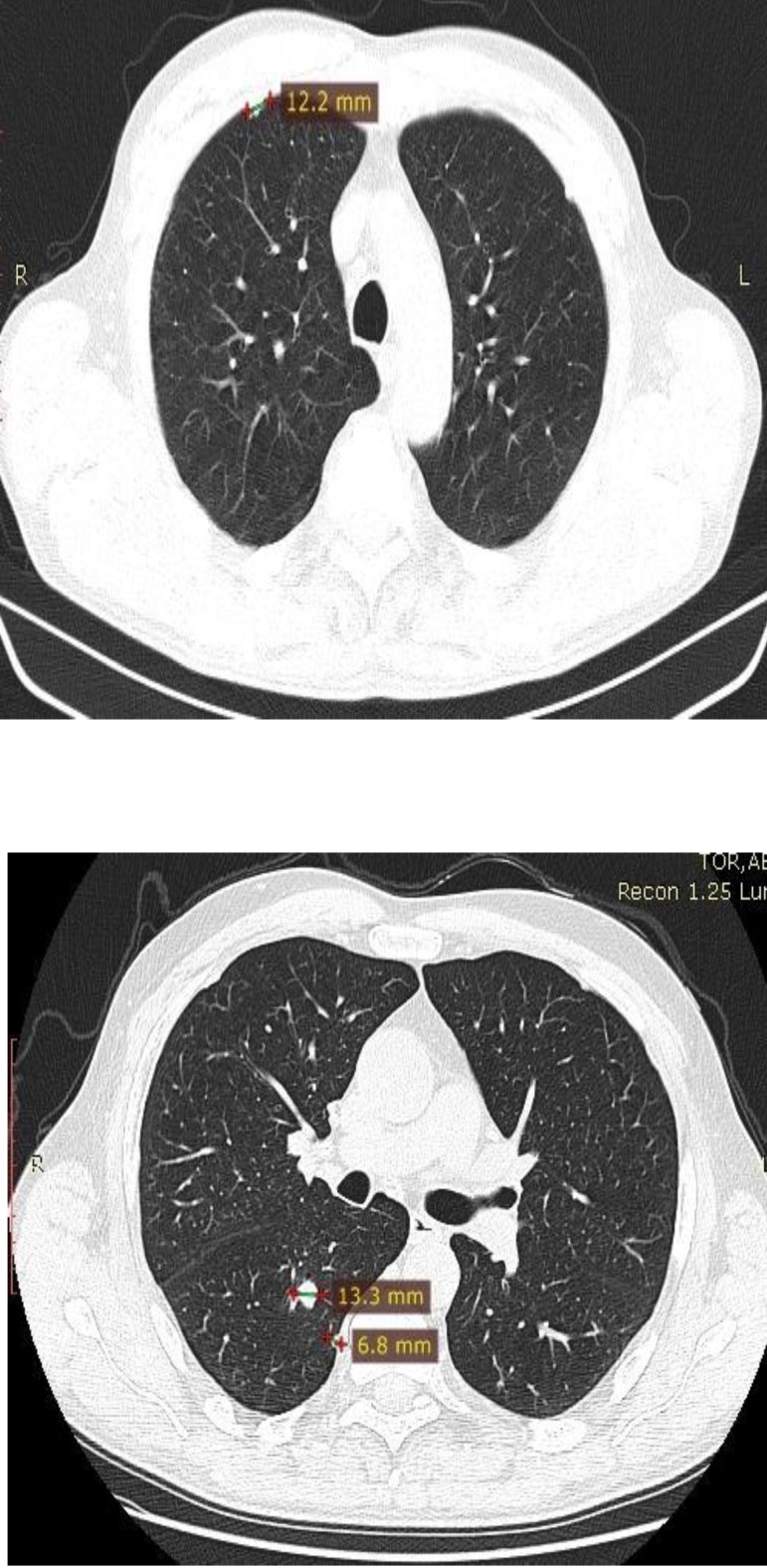
CT scan: disease progression with pulmonary metastases increased in size

Between March 2012 and September 2012, the patient received tyrosine kinase inhibitor, Sunitinib 50 mg daily 4w/ 2w, which were well tolerated. The CT scan performed in September 2012 showed a stable disease with no change in the size or number of the pulmonary metastases. The patient did not experience any side effects with Sunitinib treatment. In January 2013, a routine CT revealed a disease progression with pulmonary metastases, which increased in size (**Fig. 5**).

**Fig. 6:**
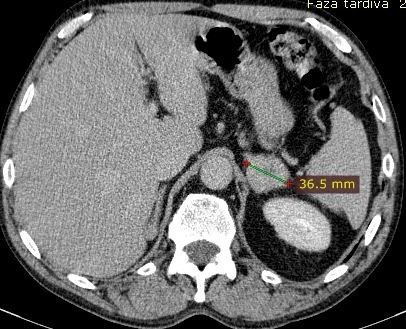
Adrenal metastasis

An adrenal metastasis was observed (**Fig. 6**). 

**Fig. 7:**
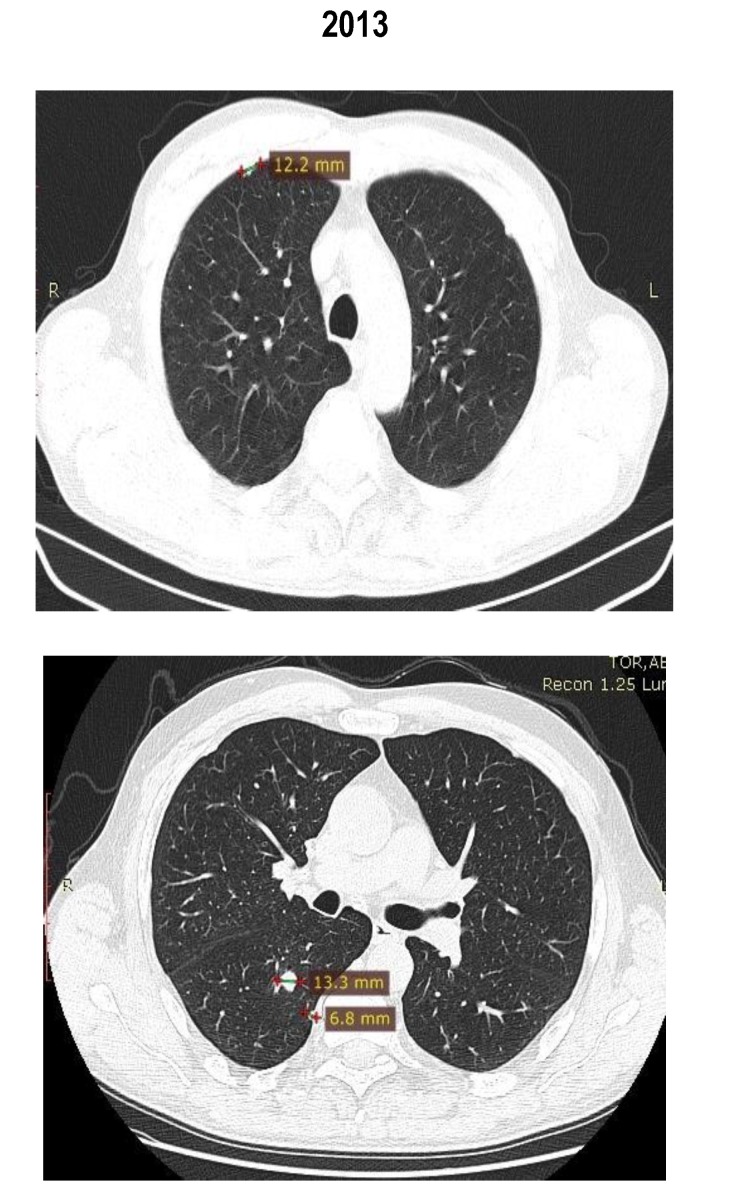
Thoracic CT scan

**Fig. 8:**
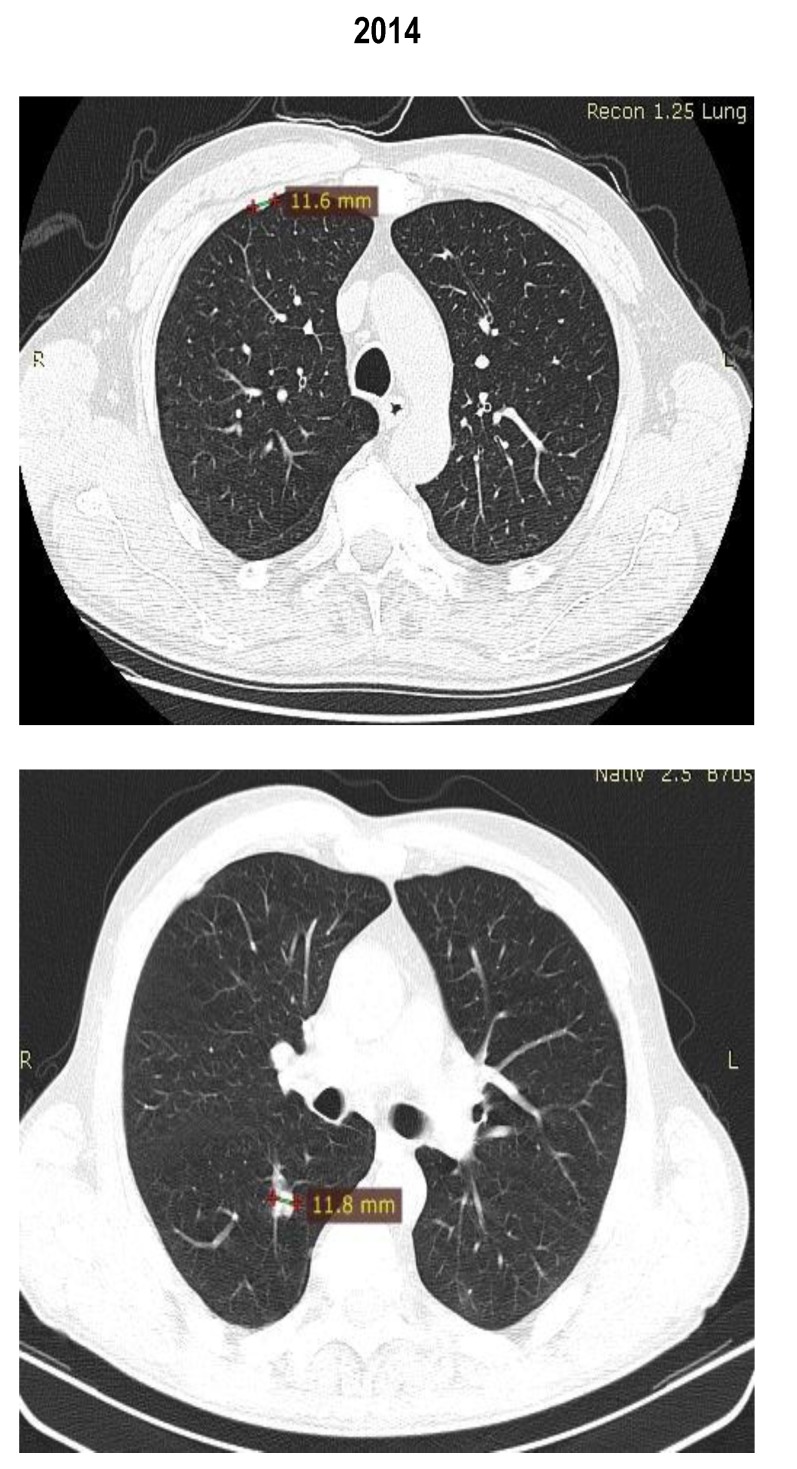
Thoracic CT scan

Between February 2013 and May 2014, the patient received treatment with mTOR inhibitor temsirolimus. In May 2014, the routine CT scan revealed a shrinkage of the lung and the adrenal metastases and the patient was considered to have a partial response (by RECIST criteria) (**Fig. 7**,**8**). The patient did not experience any side effects.

**Fig. 9:**
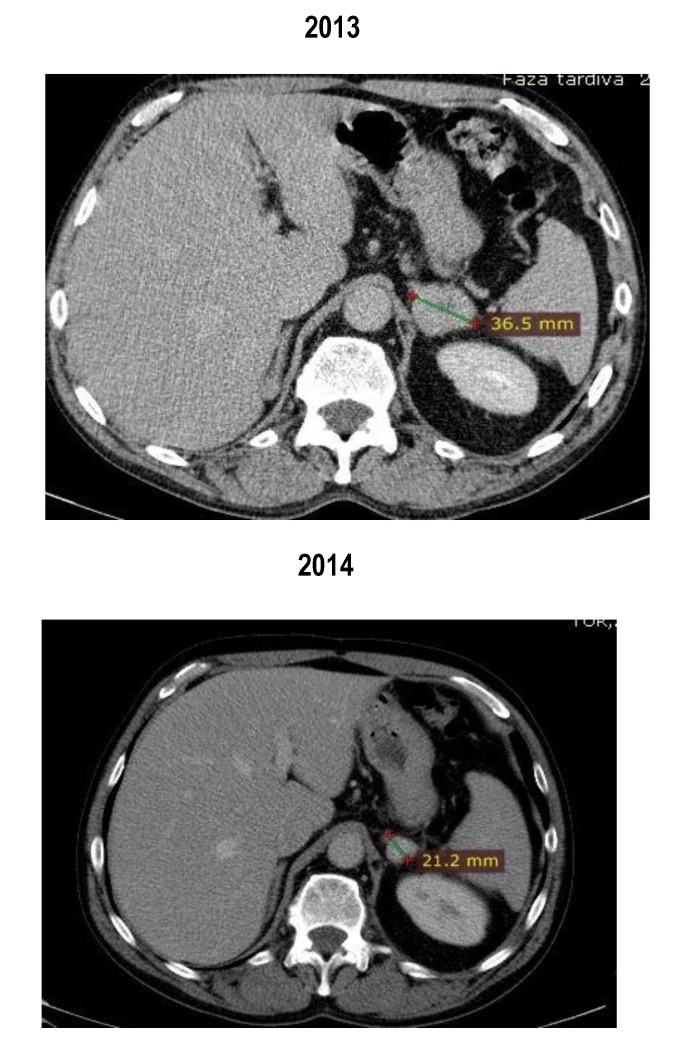
Abdominal CT scan

The images showed a partial response with shrinkage of the pulmonary lesions and suprarenal mass (**Fig. 9**).

## Discussions

With the new-targeted agents for the treatment of advanced or metastatic renal cell carcinoma (RCC), the prognosis for this condition shifts toward that of a chronic treatable disease. Six targeted agents for the treatment of advanced RCC are now approved and in clinical use: the tyrosine kinase inhibitors (TKIs) - sunitinib and pazopanib, the multikinase inhibitor - sorafenib, the anti–vascular endothelial growth factor (VEGF) monoclonal antibody - bevacizumab, and the mammalian target of rapamycin (mTOR) inhibitors - temsirolimus and everolimus. High-dose interleukin 2 (IL-2) immunotherapy and the combination of bevacizumab plus interferon-α are also approved treatments [**4**]. The novel surgical and systemic strategies have substantially changed the RCC management resulting in reduced morbidity and less invasive resection. Partial nephrectomy for small tumors and radical nephrectomy for large tumors are considered the gold standard for treatment of localized RCC. The role of metastasectomy for solitary or oligometastases presuppose long-term survival and possibly cure. Targeted therapy is the current standard of care in the treatment of metastatic RCC and/ with novel immune therapeutics being actively investigated [**5**]. The case details of a patient with mRCC treated with Sunitinib, who had a PFS for approximately 9 months, similar to the PFS observed in clinical trials, are presented. Sunitinib was also well tolerated by this patient. Temsirolimus, an mTOR inhibitor, is currently approved only as the first-line treatment of mRCC patients with poor prognosis. The treatment effect of second line temsirolimus in a patient with mRCC is demonstrated below (**Table 1**,**2**).

**Table 1:** Target therapies in the treatment of renal cancer

Treatment status	Patient status	Therapy Level 1 evidence	Other Options (≥ Level 2 )
First line Clear cell	Good or intermediate status	Sunitinib Bevacizumab + IFN-α Pazopanib	High dose IL-2 Sorafenib Observation Clinical trials
	Poor risk	Temsirolimus	Sunitinib Clinical trials
Second line Clear cell	Failed cytokines	Sorafenib Pazopanib Axitinib	Sunitinib Bevacizumab Axitinib Clinical trials
	Failed VEGEFR inhibitors	Everolimus Axitinib	Immunotherapy Gem/ 5FU TKIs Clinical trials
	Failed mTOR inhibitor	Clinical trials	
Non clear cell	Good/intermediate/poor	Clinical trial Axitinib; Bevacizumab Erlotinib Everolimus; Pazopanib Sorafenib Sunitinib; Temsirolimus	Try targeted agent

**Table 2:** ESMO Clinical Practice Guidelines 2014 for the treatment of clear cell renal tumors (Escuder B, Ann Oncol 2014; 25, Supplement 3, 1149-1156)

Treatment group	Standard	Option
2nd-line, post cytokines	Axitinib [IA] Sorafenib [IA] Pazopanib [IIA]	Sunitinib [IIA]
2nd-line, post TKIs	Axitinib [IB] Everolimus [IIA]	Sorafenib [IIA]
3rd-line post 2 TKIs	Everolimus [IIA]	
3rd-line post TKI and mTORi	Sorafenib [IB]	Other TKI [IVB] Rechallenge [IVB]

Renal cell carcinoma (RCC) includes a variety of disparate diseases, each of which displays interesting and novel molecular features, challenging some of the central tenets of cancer biology and lending unique insights into cancer-promoting mechanisms. The prevailing literature focused on the most common type, the clear cell renal cell carcinoma (ccRCC) subgroup, in which familial and sporadic disease demonstrate similar molecular profiles. ccRCC is dominated by inactivating mutations in VHL, leading to constitutive activation of the hypoxia-inducible factors (HIFs) and resultant hypoxia response transcription signature, including changes that markedly affect the cellular metabolic programs. Recent studies in ccRCC have also implicated mutations in regulators of chromatin remodeling and histone methylation [**6**]. With the development of massively parallel sequencing, a plethora of somatically mutated genes has been identified. As we now know, three genes are mutated in >10% of ccRCC, PBRM1 (mutated in ~50%), BAP1 (~15%) and SETD2 (~15%). Like VHL, all 3 genes are tumor suppressor genes. These 3 genes are placed on the short arm of chromosome 3p that encompasses VHL and is deleted in over 90% of clear cell RCC. PBRM1 mutations tend to anti-correlate with BAP1 mutations in ccRCC, and that PBRM1- and BAP1-mutated tumors exhibit a different biology and are associated with markedly different outcomes. These genes can become novel target therapies in clear cell renal cancer [**7**]. The systemic management of RCC has been maturing in recent years, with the development of a slew of rationally targeted therapies focusing on the inhibition of the VEGF and mTOR pathways. The molecular signaling pathway of PI3K/AKT/mTOR has been regularly implicated in RCC, 26% of cases had mutations involving the PI3K-AKT-mTOR pathway. Once activated, mTOR affects cell growth, proliferation, angiogenesis, and metabolism. PD-1 and PD-L1 inhibitors are a new class of agents targeting cancer cells via an immune modulated mechanism (“immune checkpoint blockade”) [**8**]. The PD-1 pathway is an important tumor-evasion mechanism, with the two principal components of the PD-1 pathway comprising PD-1 (CD279), an inhibitory receptor expressed on the surface of activated T cells, B cells and myeloid cells and PD-L1, which is expressed on cancer cells. When PD-1 and PD-L1 are bound in a complex, T cell proliferation and survival is inhibited. Conversely, PD-1 blockade leads to an auto-reactive T cell formation. PD-L1 expression has been associated with a poor prognosis in both primary and metastatic RCC. Thus, targeting either PD-1 or PD-L1 may stimulate the immune system and enhance tumor-specific cytotoxicity of T-cells. Currently, the anti-PD-1 inhibition with Nivolumab (BMS-936558) has already been evaluated in a Phase III study in metastatic RCC (NCT01668784). The American Food and Drug Administration (FDA) recently approved Nivolumab in the treatment of metastatic renal cancer [**9**].

Temsirolimus and Everolimus are two mTOR inhibitors, which proved to be active in mRCC but have never been compared in second line therapy in a prospective trial.

Iacovelli R. compared Everolimus with Temsirolimus as second-line therapy in mRCC, and reported a significant difference between the mTOR inhibitors. The results of this study indicated that Everolimus decreased the risk of death by 26% over Temsirolimus [**10**]. These results need to be confirmed in a prospective trial.

The necessity to treat the patient was sustained by a phase II study presented by Michele M. at ASCO 2012, which was designed to compare the efficiency and safety of Temsirolimus in second line treatment in metastatic renal cancer after first line with Sunitinib. The results were favorable and Temsirolimus proved to be an active and well-tolerated treatment for these patients [**11**].

## Conclusions

Despite the fact that Temsirolimus is not indicated in the second line of therapy in metastatic clear cell carcinoma according to the guidelines but also to the results of the phase II study mentioned before, this mTOR inhibitor was used after Sunitinib in a patient with metastatic clear renal cell carcinoma. The choice of treatment was constrained by the absence of other mTOR inhibitors reimbursed for metastatic renal cancer in Romania. The results were in favor of the patient with a partial response of the disease after 1 year of treatment. Temsirolimus was well tolerated the conclusion being that it can represent an option in second line treatment of patients with metastatic renal cancer when there is no other available option. Subsequent studies on a larger number of patients need to be performed to confirm our results. 
